# The diagnosis and management of adult ADHD in HMP Elmley, a Category B remand prison

**DOI:** 10.1192/bjo.2021.274

**Published:** 2021-06-18

**Authors:** Kathleen McCurdy, Nosa Igbinomwanhia

**Affiliations:** Oxleas NHS Foundation Trust, London

## Abstract

**Aims:**

Attention deficit hyperactivity disorder (ADHD) is a highly prevalent disorder in young adult prisoners. This audit aimed to identify how many residents are prescribed medication treatment for ADHD in HMP Elmley and whether those seen by the prison psychiatrists have been managed in line with NICE guidelines. We also audited waiting times and time to follow-up appointments. This was done with the overall aim to identify potential areas for development.

**Method:**

We performed a spot audit of all residents in HMP Elmley who were prescribed ADHD medication on 4th November 2019, using their electronic patient records. Appointments with the psychiatrists were then subdivided into initial assessments and follow-up appointments for the purpose of analysis. Performance was measured against NICE Guideline [NG87]: Attention deficit hyperactivity disorder: diagnosis and management. We also calculated the waiting times for initial appointment and follow-up appointment.

**Result:**

We found that 33 of residents were on ADHD medication at the time of the audit, approximately 3% of the prison population. 64% of those had a pre-existing diagnosis and 36% had been given a new diagnosis at HMP Elmley. Of those newly diagnosed 100% had undergone a Diagnostic Interview for Adults in ADHD (DIVA) assessment for diagnosis.

Baseline physical health checks had been performed in 68% of patients prior to starting medication and a cardiovascular examination had occurred in 9%. At follow-up 100% of patients had their physical observations and weight checked and their symptoms reviewed.

91% of patients were started on methylphenidate or lisdexamfetamine as first line treatment, with the rest started on atomoxetine and the reason for this documented.

100% patients were offered general psychological support.

There was a mean 22 day wait for an initial appointment (range 0-65) and a mean 20 day wait from starting medication to a psychiatric follow-up appointment (range 8-37)

**Conclusion:**

The number of residents treated for ADHD in HMP Elmley is relatively low (3%) compared to the estimated prevalence in prison population.

The key areas for improvement are in baseline cardiovascular examinations and physical health evaluations. The waiting time between initial psychiatric appointment and follow-up is another area where improvement is needed and this will form the basis of a quality improvement project.

Future steps include setting up a specific ADHD clinic with an allocated nurse practitioner to support, producing a template for ADHD assessments and follow-ups, producing a local policy on ADHD and developing specific resources for ADHD psychoeducation.

